# Supplementary material for: Phylogenetic and Comparative Analyses of Complete Chloroplast Genomes of Chinese Viburnum and Sambucus (Adoxaceae)
Source: Plants (Basel). 2020 Sep 3;9(9):1143. doi: 10.3390/plants9091143 (PMC7570041; doi:10.3390/plants9091143)
Supplement: Supplementary file 1 [file plants-09-01143-s001.zip › Supplementary files/Table S2 primers.doc]

**Table S2**. Information of the specific primer pairs used to verify the four junctions between IRs and SSC/LSC for *Viburnum* and *Sambucus*, respectively.

| Primer | Primer Sequence | Primer Length | Junction |
| --- | --- | --- | --- |
| *Viburnum* | | | |
| 1F | GCCCGGGACCACGTTATTAT | 20 | LSC/IRA |
| 1R | TTGATTCTTCGTCGCCGTAGT | 21 | LSC/IRA |
| 2F | GTCGGACAAGTGGGGAATGT | 20 | IRA/SSC |
| 2R | TTCTTCCTAGCTGCTTGGCC | 20 | IRA/SSC |
| 3F | GGAACAAGAGGGATCCACCG | 20 | SSC/IRB |
| 3R | GGCGGACGCATCTCTTCTTA | 20 | SSC/IRB |
| 4F | ACCAAGAAGAAGGATACCGTGA | 22 | IRB/LSC |
| 4R | GGATGGCCTTGTCCACACTA | 20 | IRB/LSC |
| *Sambucus* | | | |
| 5F | GCCCGGGACCACGTTATTAT | 20 | LSC/IRA |
| 5R | AGGAGTAAGCTGTGACACGTT | 21 | LSC/IRA |
| 6F | GGAACAAGAGGGATCCACCG | 20 | IRA/SSC |
| 6R | GGGTAAGACGATTGGCCGAA | 20 | IRA/SSC |
| 7F | TGTTCCACGATATGACCCATTCA | 23 | SSC/IRB |
| 7R | GGATGGCCTTGTCCACACTA | 20 | SSC/IRB |
| 8F | GTCGGACAAGTGGGGAATGT | 20 | IRB/LSC |
| 8R | TTCTTCCTAGCTGCTTGGCC | 20 | IRB/LSC |
